# Risk Factors for Unexpected Admission Following Outpatient Rotator Cuff Repair: A National Database Study

**DOI:** 10.7759/cureus.40536

**Published:** 2023-06-16

**Authors:** Salvatore J Sclafani, Matthew J Partan, John M Tarazi, Alain E Sherman, Gus Katsigiorigis, Randy M Cohn

**Affiliations:** 1 Department of Orthopaedic Surgery, Donald and Barbara Zucker School of Medicine at Hofstra/Northwell, Hempstead, USA; 2 Department of Orthopaedic Surgery, Northwell Health—Huntington Hospital, Huntington, USA; 3 Department of Orthopaedic Surgery, Northwell Health—Lenox Hill Hospital, Manhattan, USA; 4 Department of Orthopaedic Surgery, Northwell Health—Long Island Jewish Valley Stream, Valley Stream, USA

**Keywords:** unplanned readmission, national database, risk factors, 30 day readmissions, arthroscopic rotator cuff repair

## Abstract

Introduction

Rotator cuff repair (RCR) procedures are some of the most common orthopaedic surgeries performed in the United States. Compared to other orthopaedic procedures, RCRs are of relatively low morbidity. However, complications may arise that result in readmission to an inpatient healthcare facility. The purpose of this study is to identify the demographics and risk factors associated with unplanned 30-day readmission after RCR.

Methods

The American College of Surgeons National Surgical Quality Improvement Program (ACS-NSQIP) database was used to identify patients that underwent elective RCR from 2015-2019. Univariate and multivariate analyses were utilized to assess patient demographics, comorbidities, and peri-operative variables predicting unplanned 30-day readmission.

Results

Of the identified 45,548 patients that underwent RCR, 597 (1.3%) required readmission within 30 days of the procedure. Multivariate analysis identified male sex (OR 1.36, 95% CI: 1.10, 1.67), hypertension (OR 1.29, 95% CI:1.03, 1.62), chronic obstructive pulmonary disease (COPD) (OR 2.07, 95% CI: 1.46, 2.93), American Society of Anesthesiologists (ASA) Class III (OR 1.85, 95% CI: 1.07, 3.18), ASA Class IV (OR 5.38, 95% CI: 2.70, 10.72), and total operative time (OR 1.002, 95% CI: 1.000, 1.004) as independent risk factors for unplanned readmission.

Conclusion

Unplanned 30-day readmission after RCR is infrequent. However, certain patients may be at increased risk for unplanned 30-day admission to an inpatient facility. This study confirmed male sex, COPD, hypertension, ASA Class III, ASA Class IV, and total operative time to be independent risk factors for readmission following outpatient RCR.

## Introduction

Rotator cuff repair (RCR) procedures are some of the most common orthopaedic surgeries performed in the United States, with approximately 250,000 RCRs performed each year [1]. The volume of RCRs has increased substantially over the last few decades, with a major shift away from open to arthroscopic technique and routine performance in the outpatient ambulatory setting [2-5]. In general, compared to other orthopaedic procedures, RCRs have a relatively low complication rate of up to 10.6% [6-8]. Common complications reported after RCR are postoperative stiffness, hardware-related complications, and failure of repair [7,9,10].

Occasionally, complications may arise that result in unplanned 30-day readmission to an inpatient facility, with rates reported up to 1.6% [7,8,11-18]. Reported complications requiring unplanned 30-day readmission include surgical site infections, postoperative pain, and medical complications such as deep venous thrombosis and pulmonary embolism [11,13]. Previous literature has been inconclusive on risk factors for complications and readmissions after RCR. The purpose of this study is to identify the demographics and risk factors that are associated with admission to an inpatient facility within 30 days of RCR. 

## Materials and methods

Database

This study utilized the American College of Surgeons National Surgical Quality Improvement Program (ACS-NSQIP) database. Data were collected by trained clinical reviewers from over 700 participating hospitals and included patient demographics, comorbidities, surgery in Current Procedural Terminology (CPT) codes, diagnoses in International Classification of Disease 9th and 10th (ICD-9, ICD-10, respectively) revision codes, and 30-day post-operative surgical outcomes.

Patient population

The ACS-NSQIP was queried for patients who underwent RCR from 2015 to 2019 using CPT codes 23410, 23412, 23420, and 29827, yielding 60,008 cases. Patients with incomplete data were excluded (n = 14,460), resulting in a study population of 45,548 patients. CPT codes 23410, 23412, and 23420 were identified as open RCR procedures, and 29827 signified arthroscopic RCR procedures.

Variables collected

The following demographic, lifestyle, and comorbidity variables were recorded: age, sex, body mass index (BMI), American Society of Anesthesiologists (ASA) classification, race, Hispanic ethnicity, bleeding disorders, chronic obstructive pulmonary disease (COPD), diabetes mellitus, hypertension, congestive heart failure, tobacco use, and chronic steroid use. Perioperative variables that were collected included anesthesia type (general versus regional), total operative time, and procedure type (open versus arthroscopic). The primary outcome of 30-day readmission was defined as unplanned hospital readmission likely related to the principal procedure.

Statistical analyses

All data were analyzed using the Statistical Package for the Social Sciences (SPSS) version 23.0 (IBM Corp., Armonk, NY). The criterion for statistical significance was set at α = 0.05. Independent-sample student’s t-tests, chi-square test, and, where appropriate, Fisher’s exact tests were used in univariate analyses to identify demographic, lifestyle, and peri-operative variables related to 30-day readmission following RCR. Multivariate logistic regression modeling was subsequently performed. Odds ratios (ORs) with 95% confidence intervals (CIs) were calculated and reported.

## Results

Of the 45,548 patients included in our sample, 597 were readmitted within the 30-day postoperative period, corresponding to a readmission rate of 1.3%. There were 36,249 arthroscopic RCRs and 9,299 open RCRs. Demographic, lifestyle, comorbidity, and perioperative factors are presented in Table 1. The mean age of the non-admitted cohort was 58.63 ± 10.8 versus the mean age of the admitted cohort of 61.91 ± 10.4 (p < 0.001). Moreover, the mean BMI of the non-admitted cohort was 30.91 ± 6.3 versus the admitted cohort of 32.14 ± 6.9 (p <0.001).

**Table 1:** The relationship between demographic, lifestyle, comorbidity, and perioperative factors and readmission following rotator cuff repair *American Society of Anesthesiologists

	Not Admitted (N = 44,951)		Admitted (N = 597)		p-value
Outcome	N	Percent (%)	N	Percent (%)	
Age					< 0.001
<65	31,258	98.9	346	1.1	
65-79	12,928	98.3	230	1.7	
80+	560	97.2	16	2.8	
Sex					0.006
Male	26,256	98.6	382	1.4	
Female	18,694	98.9	215	1.1	
Body Mass Index, kg/m^2^					< 0.001
Normal weight	6,824	98.9	75	1.1	
Overweight	15,737	98.9	180	1.1	
Obese, Class I	12,087	98.6	172	1.4	
Obese, Class II	5,880	98.7	77	1.3	
Obese, Class III	3,851	97.9	82	2.1	
ASA* Classification					< 0.001
Class I	4,044	99.4	24	0.6	
Class II	25,466	99.1	240	0.9	
Class III	14,930	98.0	305	2.0	
Class IV	467	94.3	28	5.7	
Class V	0	0.0	0	0	
Race					0.288
White	32,811	97.2	543	1.4	
Black or African American	3,548	98.4	59	1.6	
Asian	1,130	98.6	16	1.4	
American Indian/Alaskan Native	346	98.3	6	1.7	
Native Hawaiian/Pacific Islander	206	97.2	6	2.8	
Hispanic	3,569	99.3	26	1.7	< 0.001
Bleeding Disorder	443	96.7	15	3.3	< 0.001
Chronic Obstructive Pulmonary Disease	1,475	96.4	55	3.6	< 0.001
Diabetes	7,552	98.0	156	2.0	< 0.001
Hypertension	20,847	98.3	369	1.7	< 0.001
Congestive Heart Failure	57	89.1	7	10.9	< 0.001
Steroid Use	880	97.7	21	2.3	0.007
Current Smoker	7,059	98.4	117	1.6	0.009
Anesthesia Type					0.073
General	586	99.7	2	0.3	
Regional	19,476	98.0	221	1.1	
Total Operative Time	44,949	85.65 ± 45.3	597	90.1 ± 46.6	0.017
Procedure Type					0.001
Arthroscopic	35,806	98.8	443	1.2	
Open	9,145	98.3	154	1.7	

The results of the univariate analysis revealed statistically significant relationships between readmission status and the tested patient variables are itemized in Table 2.

**Table 2:** Univariate analysis between readmission status and patient variables *American Society of Anesthesiologists

Variable	Test (Interval)	p-Value
Patient age	χ^2^ (2, 45,338) = 40.58	< 0.001
Sex	χ^2^ (1, 45,547) = 7.54	0.006
Body Mass Index (BMI)	χ^2^ (4, 44,965) = 25.82	< 0.001
ASA* Classification	χ^2^ (3, 45,504) = 172.98	< 0.001
Hispanic Ethnicity	χ^2^ (1, 37,135) = 13.78	< 0.001
Bleeding Disorder	χ^2^ (1, 33,337) = 13.00	< 0.001
Chronic Obstructive Pulmonary Disease	χ^2^ (1, 45,548) = 63.85	< 0.001
Diabetes	χ^2^ (1, 45,548) = 36.48	< 0.001
Hypertension	χ^2^ (1, 45,548) = 56.39	< 0.001
Congestive Heart Failure	χ^2^ (1, 45,548) = 45.92	< 0.001
Steroid Use	χ^2^ (1, 45,548) = 7.39	0.007
Current Smoker	χ^2^ (1, 45,548) = 6.73	0.009
Total Operative Time	t(45,546) = 2.38	0.017
Procedure Type	χ^2^ (1, 45,548) = 10.78	0.001

Patient race and anesthesia type were not significantly associated with readmission.

Multivariate logistic regression modeling confirmed that the following patient variables were associated with statistically significantly increased odds of readmission (see Table 3): sex, p = 0.004, OR 1.36, 95% CI (1.10, 1.67); hypertension, p = 0.029, OR 1.29, 95% CI (1.03, 1.62); ASA Class III, p = 0.027, OR 1.85, 95% CI (1.07, 3.18); ASA Class IV, p < 0.001, OR 5.38, 95% CI (2.70, 10.72); total operative time, p = 0.048, OR 1.002, 95% CI (1.000, 1.004); COPD, p < 0.001, OR 2.07, 95% CI (1.46, 2.93). Hispanics were associated with statistically significant decreased odds of readmission, p = 0.018, OR 0.59, 95% CI (0.38, 0.92). The relationship between age, BMI, race, bleeding disorder, diabetes, CHF, steroid use, current smokers, procedure type, and readmission did not achieve statistical significance in the multivariate model.

**Table 3:** Multivariate analysis related to outcomes of interest *American Society of Anesthesiologists

Outcome	OR	CI	p-Value
Age (reference = <65)			< 0.001
65-79	1.36	95% CI (0.33, 5.59)	0.668
80+	1.97	95% CI (0.43. 9.09)	0.384
Sex (reference = male)	1.36	95% CI (1.10, 1.67)	0.004
Hispanic	0.59	95% CI (0.38, 0.92)	0.018
Hypertension	1.29	95% CI (1.03, 1.62)	0.029
ASA* (reference = Class I)			< 0.001
Class II	0.98	95% CI (1.03, 1.62)	0.952
Class III	1.85	95% CI (1.07, 3.18)	0.027
Class IV	5.38	95% CI (2.70,10.72)	< 0.001
Total Operative Time	1.002	95% CI (1.000, 1.004)	0.048
Chronic Obstructive Lung Disease	2.07	95% CI (1.46, 2.93)	< 0.001

## Discussion

Rotator cuff repairs are one of the most common orthopaedic surgeries performed in the United States, with approximately 250,000 performed each year. Although RCRs are generally of low morbidity, complications may arise that result in unplanned 30-day readmission to an inpatient facility [7,8,11-18]. The goal of our study was to utilize the ACS-NSQIP database from 2015-2019 to evaluate risk factors for patient readmission within 30 days following outpatient RCR. During the study period, the readmission rate within 30 days of surgery was 1.3%. Previous studies have shown comparable readmission rates of approximately 1.6% [7,8,11-18]. Our analysis revealed statistically significant relationships between readmission status and the following patient variables: patient age, male sex, BMI, ASA classification, Hispanic ethnicity, bleeding disorder, COPD, diabetes, hypertension, steroid use, current smoker, total operative time, and procedure type. When accounting for all related outcomes of interest, our multivariate logistic regression modeling identified male sex, total operative time, COPD, hypertension, and ASA Class III and IV to be independent risk factors for unplanned 30-day readmission. Interestingly, the relationship between age, BMI, race, bleeding disorder, diabetes, CHF, steroid use, current smokers, procedure type (open versus arthroscopic), and readmission did not achieve statistical significance in the multivariate model. Additionally, our study determined that Hispanic ethnicity was associated with lower odds of readmission after RCR. These findings can provide orthopaedic surgeons and other pertinent providers with knowledge of potentially modifiable risk factors for patients undergoing RCR.

Multiple studies have used the ACS-NSQIP database from previous years to evaluate for risk factors for complications and unplanned readmissions associated with RCR. A study by Heyer et al. utilized the ACS-NSQIP database to evaluate risk factors for 30-day complications following arthroscopic RCR [15]. The authors analyzed 21,143 patients from 2006-2015 and found ASA Class > II, history of COPD, and dyspnea to be risk factors for complications. The most common complication reported during this study was a venous thromboembolic event. However, this study did not assess for risk factors associated with 30-day readmission after RCR [15]. Day et al. queried the ACS-NSQIP database from 2005-2013 to compare short-term complications after open versus arthroscopic RCR [18]. The overall complication rate reported was 1.3%. They identified age greater than 65, operative time greater than 90 minutes, and open repair as risk factors for complications. The authors also reported a 30-day readmission rate of 1.16% [18]. Schairer et al. and Hill et al. used the ACS-NSQIP dataset to identify risk factors for complications and unplanned hospital readmission within 30 days of RCR [13,16]. Schairer et al. demonstrated open repair, male gender, increased age, and medical comorbidities to be associated with a significantly increased risk of complications and hospital readmission [16]. Hill et al. found an overall readmission rate of 0.98%, with the most common reason being pulmonary embolism [13]. The authors found operative time >1.5 hours, age >40 years, ASA Class III or IV, and chronic steroid use as risk factors for readmission following arthroscopic shoulder surgery [13].

Reports of gender as a risk factor for readmission have been variable. While some reports, including the current study, demonstrate male sex is associated with an increased risk of complications and readmission, others have identified female sex to be associated with a higher rate of complications [7,15,16,19]. Gil et al. found female sex to be a significant predictor of admission after outpatient surgery [20]. On the other hand, while Kosinski et al. did not find an increased risk of readmission for male sex, they determined female sex to be associated with lower odds of admission [17]. In contrast, our findings suggested that male sex had higher odds of readmission after RCR.

Increased BMI and the presence of medical comorbidities, including COPD, hypertension, chronic steroid use, dialysis, and metastatic cancer, have also been reported to be associated with an increased rate of complications and unplanned readmission after RCR [12,13,15,16,19-21]. In addition, several authors have demonstrated higher ASA Class > II to be a significant risk factor for complication and readmission [12,13,15,19,20]. The current study found patients with comorbidities, such as COPD and hypertension, to have an increased risk of readmission within 30 days of RCR. The current study also found ASA Class III and IV to be risk factors for readmission within 30 days of RCR. However, we did not find an association between CHF, diabetes, bleeding disorder, or steroid use with an increased risk of admission within 30 days of surgery.

Smoking has long been reported as a risk factor for complications following the repair of rotator cuff tears [14,16,18]. Best et al. used the ACS-NSQIP database from 2011-2016 to evaluate 5,157 patients undergoing open rotator cuff repair [22]. The authors determined that smokers are at increased risk of short-term complications, including venous thromboembolism and pulmonary embolism [22]. In a study by Kashanchi et al., the authors investigated the association between smoking status and postoperative complications within 30 days of arthroscopic RCR [23]. They demonstrated smoking to be a significant predictor of surgical complications, return to the operating room, readmission, and sepsis or septic shock [23]. Smoking was not identified as an independent risk factor for readmission after RCR in our multivariate logistic regression model.

Longer operative times have been previously recognized as a risk factor for complications after various orthopaedic procedures, including shoulder arthroscopy, anterior cruciate ligament reconstruction, and total joint arthroplasty, among others [13,18,19,24-26]. Day et al. and Hill et al. found an increased risk of complications and 30-day readmission, respectively, with operative times > 90 minutes [13,18]. Boddapati et al. specifically examined shoulder arthroscopy procedure time and its effect on the rates of short-term postoperative complications, readmissions, and overnight hospital stays [19]. The authors identified an increased risk of superficial surgical site infections and overnight hospital stay for procedures lasting between 45 minutes and 90 minutes, and for procedures lasting greater than 90 minutes, when compared with procedures that were less than 45 minutes [19]. Similarly, our study demonstrated increased odds of readmission within 30 days of RCR with higher total operative times.

Lastly, our study did not demonstrate a significantly increased odds of admission when comparing open versus arthroscopic techniques. However, Baker et al. utilized the ACS-NSQIP database from 2007-2014 to provide a comparative report of complications and unplanned readmission rates after open and arthroscopic RCR [21]. The authors determined that patients undergoing open RCR had a higher risk of adverse events, as well as a higher risk of a return to the operating room within 30 days when compared to the arthroscopic group. Open RCR was also associated with a longer average hospital stay. It is important to note that the open RCR group had a higher prevalence of patients aged 65 or older, and comorbidities such as hypertension, diabetes, COPD, smoking, and alcoholism [21]. Owens et al. compared 6,975 open rotator cuff repairs and 2,918 arthroscopic rotator cuff repairs [14]. The authors demonstrated the arthroscopic group had a significantly lower risk of complications, a lower rate of superficial infection, a lower incidence of return to the operating room within 30 days, and a lower risk of hospital readmission [14]. Moreover, several of the previously mentioned studies have also demonstrated increased rates of complications and readmission with the open technique [16,18,20].

The authors included 45,548 patients in this study. Despite the large number of cases available for review, there are some limitations to the current study. Inherent limitations to the NSQIP database are potential coding errors and misclassifications, which may lead to incomplete patient capture. However, inter-rater reliability disagreement within the database has been shown to be low, at less than 1.8% [27]. Furthermore, the ACS-NSQIP database is reliant on reports of participating hospitals. Therefore, there is potential that the data may only reflect the population of the participating institutions, rather than a much larger population. However, as the number of participating institutions increases, the data extrapolated may be better applied to a larger population.

## Conclusions

The current study analyzed a large sample of patients that underwent RCR and evaluated for risk factors associated with unplanned 30-day admission. Our analysis revealed statistically significant relationships between readmission status and the following patient variables: patient age, sex, BMI, ASA classification, Hispanic ethnicity, bleeding disorder, COPD, diabetes, hypertension, steroid use, current smoker, total operative time, and procedure type. Multivariate logistic regression modeling confirmed male sex, COPD, hypertension, ASA Class III and IV, and total operative time to be independent risk factors for readmission following outpatient RCR. Such findings can allow the orthopaedic surgeon to identify patients at higher risk for readmission preoperatively, discuss expectations with their patients, and reduce costs by preventing avoidable complications.
